# A Rare Case of Gastroparesis-Induced Takotsubo Cardiomyopathy

**DOI:** 10.7759/cureus.64636

**Published:** 2024-07-16

**Authors:** Joane Titus, Jacob Bentley, Kristin L Recker, Olga Karasik

**Affiliations:** 1 Internal Medicine, University of Central Florida (UCF) Hospital Corporation of America (HCA) Graduate Medical Education (GME) Consortium, Orlando, USA; 2 Internal Medicine, University of Central Florida (UCF) College of Medicine, Orlando, USA

**Keywords:** atypical takotsubo cardiomyopathy, gastrointestinal causes of takotsubo, gastroparesis induced takotsubo cardiomyopathy, gastroparesis, takotsubo cardiomyopathy (tcm)

## Abstract

This case report includes an extremely rare and intriguing presentation of takotsubo cardiomyopathy (TCM), triggered by forceful vomiting in the setting of gastroparesis, a condition characterized by delayed gastric emptying. TCM is a reversible form of cardiomyopathy that typically occurs following a severe emotional or physical stressor. In this exceptional case, we present a patient with an acute severe episode of gastroparesis, followed by TCM, devoid of any recognizable emotional or physical stressors. This case highlights the importance of considering non-traditional triggers in TCM cases.

## Introduction

Takotsubo cardiomyopathy (TCM), also known as stress-induced cardiomyopathy or "broken heart syndrome," is a reversible form of heart muscle dysfunction characterized by the sudden onset of left ventricle apical hypokinesia, akinesia, or dyskinesia [1]. TCM is typically associated with severe emotional or physical stressors, such as the death of a loved one, a natural disaster, or a medical illness [1]. However, the onset of TCM secondary to gastrointestinal (GI) disorders like gastroparesis is very rare and has not yet been documented. We present a case of gastroparesis-induced TCM to highlight this uncommon clinical association.

## Case presentation

**Figure 1:**
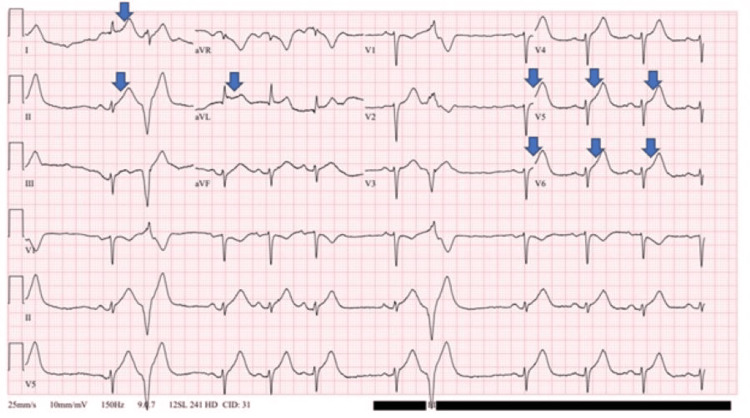
EKG on initial presentation demonstrating sinus arrhythmia with ST elevation in the inferolateral leads indicated by arrows, with occasional PVCs EKG: Electrocardiogram; PVCs: Premature ventricular complexes

Our patient was a 45-year-old woman with a medical history of non-insulin-dependent diabetes mellitus and gastroparesis, on ondansetron and metoclopramide. She presented to the Emergency Department due to persistent nausea and vomiting, along with chest tightness, diaphoresis, palpitations, shortness of breath, abdominal discomfort, and chills for three days. She reported forceful vomiting around 10-15 times per day prior to admission. On arrival, her vitals included a blood pressure of 198/88 mmHg, an initial troponin of 518 ng/L followed by a second troponin of 3443 ng/L two hours later. Her initial electrocardiogram (EKG) demonstrated a heart rate of 69 bpm with marked sinus arrhythmia and occasional premature ventricular complexes (PVCs), in addition to ST elevations in leads I, II, aVL, V5, and V6, suggestive of acute inferolateral myocardial infarction or injury (Figure 1).

Due to the acute onset of shortness of breath, chest discomfort, and GI symptoms, both computed tomography angiography (CTA) and computed tomography (CT) of the abdomen were obtained. CTA was negative for pulmonary embolus, and the CT of the abdomen was negative except for an incidental finding of subaortic stenosis. Aspartate aminotransferase (AST) was 33 units/L, alanine aminotransferase (ALT) was 13 units/L, and alkaline phosphatase was 49 units/L. An echocardiogram showed an ejection fraction of 30-35% with mild to moderate dilation of the left atrium, mild mitral regurgitation, no pericardial effusion, and most notably, apical ballooning of the apex suggestive of takotsubo syndrome (Video 1). Left heart catheterization revealed a discrete lesion in the left anterior descending artery, which did not warrant percutaneous intervention.

**Video 1:** Echocardiogram showing a reduced ejection fraction of 30-35% with apical ballooning of the apex suggestive of takotsubo syndrome

Over the course of her hospitalization, the patient's troponin peaked at 7828 ng/L approximately six hours after arrival at the hospital before trending down. Her repeat EKG again showed elevations in the inferior and lateral leads. The persistently elevated troponin level and EKG findings are not uncommon for patients with TCM. For her gastroparesis, metoclopramide was resumed at a dose of 10 mg three times daily, and ondansetron was initiated at a dose of 4 mg every six hours as needed. The patient was eventually discharged with metoclopramide 10 mg three times daily as needed and ondansetron 4 mg every eight hours as needed. During her stay, she had improvement and resolution of her chest pain, nausea, and vomiting. The final recommendations were for the patient to be sent home on goal-directed medical therapy (GDMT) and a life vest. GDMT consisted of metoprolol succinate 12.5 mg once daily, spironolactone 12.5 mg once daily, and empagliflozin 10 mg once daily. Sacubitril/valsartan was also recommended for the patient, but she was unable to afford the medication due to a lack of insurance. Within a couple of months, the patient had clinically recovered and was able to return to her baseline activity.

## Discussion

TCM is a result of severe stressors which can lead to heart dysfunction. In most cases, TCM is associated with acute emotional or physical stressors such as sepsis, trauma, or significant emotional events [1]. There have been few reported cases of TCM associated with forceful vomiting secondary to acute-on-chronic vomiting [2] and cyclical vomiting syndrome [3]. However, this is the first reported case that presents vomiting secondary to gastroparesis as a cause of TCM. The exact pathophysiological mechanisms by which forceful vomiting leads to TCM remain unclear. The prevailing hypothesis suggests that TCM results from elevated levels of plasma catecholamines and circulating metabolites due to stress [4]. Recognizing the diverse range of stressors that can precipitate TCM is crucial for clinicians. The overlap in clinical presentation between TCM and acute myocardial infarction (AMI) means that prompt and accurate diagnosis is essential to manage the condition effectively [1]. The risk of complications, such as heart failure and stroke, has been found to be higher in hospitalized TCM patients when compared to AMI patients, underscoring the importance of timely intervention [5].

Due to the risk of severe complications from TCM, it is imperative that a diagnosis be made expeditiously to identify the trigger and initiate treatment to resolve it. The incidence of TCM is about 0.02% of all hospitalizations in the United States, with an increased propensity in elderly women, smokers, alcohol abusers, and those with anxiety states [6,7]. For an official diagnosis of TCM based on the Mayo Clinic criteria, the following conditions must be met: suspicion of AMI based on the clinical presentation and ST-segment elevation noted on an EKG [6]. Then, imaging confirms ventriculography or an echocardiogram demonstrating transient hypokinesia or akinesia of the ventricle [6]. Then, heart catheterization or arteriography confirms normal coronary arteries, with less than 50% luminal narrowing in all coronary arteries [6]. Lastly, other conditions that may mimic TCM (such as myocarditis) should be excluded. In our case, this patient met all of the Mayo Clinic criteria for TCM. Her lack of recent illness, fever, or pericardial effusion helped exclude myocarditis as a likely cause. 

With a review of the literature, there were other GI-related cases of TCM. One involved an 85-year-old man who was admitted for ileus with severe abdominal pain and vomiting. Ventriculography revealed an ejection fraction of 33% with apical dilation, supporting TCM. He was treated with similar medications and discharged without significant adverse events [8]. Another GI case involved a 27-year-old man with a history of alcohol abuse who developed TCM as a complication of pancreatitis in the absence of vomiting [9]. Both cases demonstrate the need for consideration of GI conditions contributing to the development of TCM.

## Conclusions

TCM is rare, and its etiology is not well understood, but this case highlights the broad spectrum of potential causes for TCM. By detailing this atypical presentation, the authors want to emphasize the need to consider GI causes of TCM when evaluating a patient with no other risk factors and how treating gastroparesis will contribute to decreased morbidity in these patients.
